# Cost-effectiveness of a simplified acute malnutrition program: a secondary analysis of the OptiMA randomized clinical trial in the Democratic Republic of the Congo

**DOI:** 10.1093/heapol/czae106

**Published:** 2024-11-08

**Authors:** Stephen C Resch, Ryoko Sato, Kevin Phelan, Cécile Cazes, Abdramane Ombotimbe, Victoire Hubert, Harouna Boubacar, Liévin Izie Bozama, Gilbert Tshibangu Sakubu, Béatrice Kalenga Tshiala, Toussaint Tusuku, Rodrigue Alitanou, Antoine Kouamé, Cyrille Yao, Delphine Gabillard, Moumouni Kinda, Renaud Becquet, Susan Shepherd, Robert M Hecht

**Affiliations:** Center for Health Decision Science, Harvard University TH Chan School of Public Health, 677 Huntington Avenue, Boston, MA 02115, USA; Center for Health Decision Science, Harvard University TH Chan School of Public Health, 677 Huntington Avenue, Boston, MA 02115, USA; The Alliance for International Medical Action (ALIMA), 1 Rue Philidor, Paris 75020, France; Bordeaux Population Health Research Centre, University of Bordeaux, National Institute for Health and Medical Research (INSERM) UMR 1219, Research Institute for Sustainable Development (IRD) EMR 271, 146 Rue Léo Saignat 11, Bordeaux 33076, France; ALIMA, Kamuesha, Democratic Republic of the Congo; ALIMA, Kamuesha, Democratic Republic of the Congo; ALIMA, Kamuesha, Democratic Republic of the Congo; National Nutrition Programme, Ministry of Health, Kinshasa, Democratic Republic of the Congo; Kamuesha Health Zone in the Kasaï Province, Ministry of Health, Kamuesha, Democratic Republic of the Congo; National Nutrition Programme, Ministry of Health, Kinshasa, Democratic Republic of the Congo; National Nutrition Programme, Ministry of Health, Kinshasa, Democratic Republic of the Congo; ALIMA, Kamuesha, Democratic Republic of the Congo; PACCI ANRS Research Programme, University Hospital of Treichville, Abidjan, Côte d’Ivoire; PACCI ANRS Research Programme, University Hospital of Treichville, Abidjan, Côte d’Ivoire; Bordeaux Population Health Research Centre, University of Bordeaux, National Institute for Health and Medical Research (INSERM) UMR 1219, Research Institute for Sustainable Development (IRD) EMR 271, 146 Rue Léo Saignat 11, Bordeaux 33076, France; ALIMA, Rte des Almadies, Dakar, Senegal; Bordeaux Population Health Research Centre, University of Bordeaux, National Institute for Health and Medical Research (INSERM) UMR 1219, Research Institute for Sustainable Development (IRD) EMR 271, 146 Rue Léo Saignat 11, Bordeaux 33076, France; ALIMA, Rte des Almadies, Dakar, Senegal; Yale University School of Public Health, 60 College St, New Haven, CT 06510, USA

**Keywords:** cost-effectiveness, acute malnutrition, randomized clinical trial, Democratic Republic of the Congo

## Abstract

Acute malnutrition (AM) causes large loss of life and disability in children in Africa. Researchers are testing innovative approaches to increase the efficiency of treatment programs This paper presents results of a cost-effectiveness analysis of one such program in the Democratic Republic of the Congo (DRC) based on a secondary analysis of a randomized controlled trial Optimizing Treatment for Acute Malnutrition (OptiMA), conducted in DRC in 2018–20. A total of 896 children aged 6–59 months with a mid-upper arm circumference (MUAC) <125 mm or with oedema were treated and followed for 6 months. The cost-effectiveness of OptiMA using ready-to-use therapeutic food (RUTF) at a tapered dose was compared with the standard national program in which severe cases (SAM) received RUTF proportional to weight, and moderate cases (MAM) were referred to another clinic for a fixed dose regimen of ready-to-use supplementary food. Cost analysis from the provider perspective used data collected during the trial and from administrative records. Statistical differences were derived using t-tests. The mean cost per enrolled child under OptiMA was $123 [95% confidence interval (CI): 114–132], not statistically different from the standard group [$127 (95%CI: 118–136), *P* = 0.549], while treatment success (i.e. recovery to MUAC > 125 mm and no relapse for 6 months) under OptiMA was 9% higher (72 vs 63%, *P* = 0.004). Among children with SAM at enrollment, there was no significant difference in treatment success between OptiMA and standard care (70 vs 62%, *P* = 0.12), but OptiMA’s mean cost per enrolled child was 23% lower ($128 vs $166, *P* < 0.0001). OptiMA was more effective at preventing progression to SAM among those enrolled with MAM (5 vs 16%, *P* < 0.0001), with an incremental cost-effectiveness ratio of $234 per progression to SAM prevented. Overall, OptiMA had significantly better outcomes and was no more expensive than standard care. Its adoption could enable more children to be successfully treated in contexts where therapeutic food products are scarce.

Key messages

- To our knowledge, this is the first cost-effectiveness analysis of a randomized controlled trial of an integrated, simplified strategy of acute malnutrition treatment in children aged 6–59 months in the Democratic Republic of the Congo (DRC).
- We compared the cost and effectiveness of the current national standard strategy with that of the OptiMA strategy. The standard strategy has separate protocols and products for SAM and MAM management using RUTF at an increasing dose with increasing weight in children with SAM and ready-to-use supplementary food at a fixed dose in children with MAM. By contrast, the OptiMA strategy is a single protocol for both SAM and MAM using only RUTF at a decreasing dose with increasing weight.
- The cost-effectiveness analysis presented in this paper found that across all patients in the DRC trial with acute malnutrition, OptiMA was the dominant strategy (more effective at a lower cost) as compared to the national standard strategy.
- Adoption of the OptiMA approach would likely enable more children to be successfully treated at a cost similar to that of the current status quo of separate and parallel SAM and MAM treatment programs prevailing in most countries of sub-Saharan Africa and other lower- and middle-income regions of the world.

## Introduction

Malnutrition is endemic in many parts of the world today, with 149 million children <5 years old stunted and at least 45 million wasted (United Nations Children’s Fund (UNICEF), World Health Organization, International Bank for Reconstruction and Development/The World Bank 2021). The Democratic Republic of the Congo (DRC) has one of the largest malnutrition problems, because of disease burden, poor diet, and poverty, aggravated by conflict (World Food Programme 2018). UNICEF estimates that >3 million children <5 years old suffered from acute malnutrition in DRC in 2021 (United Nations Children’s Fund (UNICEF), World Health Organization, International Bank for Reconstruction and Development/The World Bank 2021).

Even though effective interventions exist, national malnutrition programs face substantial financial constraints, and thus <30% of malnourished children currently have access to treatment (World Food Programme 2018, No Wasted Lives 2019), due to a mix of supply-side and demand-side constraints. This jeopardizes the achievement of global nutrition targets for 2025 of reducing the number of stunted children to 100 million (World Health Organization 2014) and the Sustainable Development Goal of zero hunger by 2030 (United Nations Children’s Fund (UNICEF), World Health Organization, International Bank for Reconstruction and Development/The World Bank 2021). In addition to mobilizing additional resources for prevention and treatment, innovations to improve the efficiency of acute malnutrition (AM) treatment are needed to make scarce available resources go further.

The standard approach to addressing AM divides malnourished children into severe [SAM: mid-upper arm circumference (MUAC) <115 mm and/or weight-for-height z-score (WHZ) < −3, and/or presence of edema] and moderate (MAM: MUAC between 115 and 125 mm and WHZ ≥ −3) and treats them in separate programs using different nutritional products supplied by parallel supply chains, which complicates logistics and coordination of service delivery. Furthermore, treatment for MAM is endorsed by the World Health Organization (WHO) only in contexts limited to severe food insecurity or conflict and is thus often unavailable alongside SAM programming (World Health Organization 2017).

The Optimizing Treatment for Acute Malnutrition (OptiMA) trial was conducted in DRC in 2018–20 (clinical trials.gov NCT03751475) (Cazes et al. 2022, 2023). OptiMA aims to improve the efficiency of resource use and maintain the continuity and effectiveness of treatment by defining eligibility for treatment as MUAC < 125 mm or clinical detection of nutritional edema. This shortens the time taken to determine whether children are eligible for treatment and lowers resource requirements by involving only one health worker instead of the two needed to measure both MUAC and WHZ under the prevailing standard nutrition guidelines in DRC.

OptiMA uses a single product [ready-to-use therapeutic food (RUTF)] and tapers the dose as weight and MUAC increase. The standard national approach in DRC treats SAM patients with an increasing RUTF dose as the child gains weight and MAM patients with a fixed dose (500 kcal/day) of ready-to-use supplementary food (RUSF) regardless of weight (RUTF was manufactured by Nutriset, Hameau du Bois Ricard, CS 80 035–76 770 Malaunay, France, and purchased by UNICEF; RUSF was from the same manufacturer and provided by the World Food Programme). The simplified OptiMA approach, which offers a ‘one-stop shop’ for AM treatment, is designed to improve the cost-effectiveness of treatment by reducing the logistical complexity and corresponding quantity of labor and RUTF used per course of treatment, while still aiming to maintain effectiveness as measured through nutritional outcome indicators.

The primary outcome of the study was the success rate, defined as alive, not acutely malnourished, and without relapse during the 6 month period after the initiation of the program. Cazes *et al*. (2022) found that the success rate among children in the OptiMA group was 9.0% higher than that in the standard group [95% confidence interval (CI) 2.0–15.9].

In this paper, we examine the cost of treatment strategies in the trial and measure the overall cost-effectiveness of OptiMA in terms of cost per additional successfully treated case, the probability that OptiMA is cost-effective, and, among the subset of patients with MAM at enrollment, the cost per episode of progression from MAM to SAM.

## Methods

### Study design and participants

This study is a secondary analysis of a randomized controlled trial called OptiMA, conducted in the Kamuesha health zone, Kasai Province, DRC in 2019–2020. Kamuesha is a remote district of 500 000 people with 26 health centres and one district hospital. In 2019, this landlocked rural health zone experienced persistent armed conflict, population displacement, and substantial food insecurity. The malnutrition treatment project that hosted the trial was implemented by the Alliance for International Medical Action (ALIMA), a non-governmental organization (NGO). Over 7 months beginning 22 July 2019, the trial enrolled 896 children aged 6–59 months with a MUAC < 125 mm or with oedema (Cazes et al. 2022). These 896 children were only a fraction of all children admitted to the 26 health centres in the study. This is because one of the purposes of the OptiMA trial was to conduct non-inferiority analysis among SAM children, while the majority of admitted children were MAM and thus the study stopped enrolling MAM while ensuring a sufficient sample of SAM children.

### Trial design

The individual-level randomized controlled trial was launched on 1 May 2018, with children randomized 1 : 1 to either the standard or OptiMA arm. Inclusion criteria included all children living in the trial catchment area aged between 6 and 59 months with a MUAC < 125 mm, bilateral oedema, or WHZ score of < −3. Exclusion criteria were medical conditions requiring hospitalization; no appetite; grade 3 oedema; known allergy to milk, peanuts, or RUTFs; any chronic pathology; MUAC ≥125 mm with no bilateral oedema but a WHZ score < −3; and siblings of children already randomly assigned in the trial. Although children who required inpatient care at the outset were not enrolled, those who deteriorated to the point where they required inpatient care were included in the overall analysis.

The final sample sizes in the standard group and OptiMA were 446 and 450, respectively. Sex data were collected based on self-reporting and the options were either female or male. In the sample, the proportion of females was 50 and 51% for the standard group and OptiMA, respectively. Study children were further categorized into two groups according to initial nutritional status based on the WHO definition: moderate acute malnutrition (MAM: MUAC 115–125 mm and WHZ ≥ −3) and severe acute malnutrition (SAM: MUAC <115 mm and/or WHZ ≤ 3, and/or presence of edema). See Cazes *et al*. (2022)  Fig. 1 for the trial profile.

**Figure 1.**
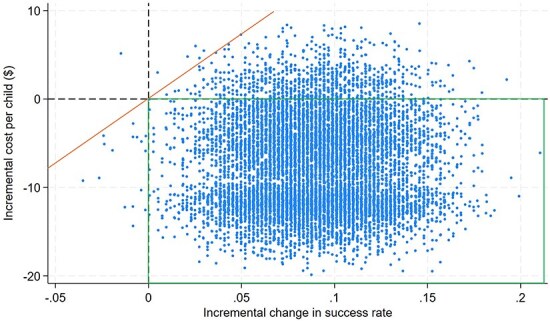
Sensitivity analysis.

Table 1 summarizes the differences in program design between the OptiMA and standard arms. For the latter, children with SAM received RUTF at a weight-based dosage with the amount increasing as the child’s weight increased. Children in the OptiMA group, regardless of nutritional status (SAM or MAM), received RUTF at a dosage that gradually decreased as the child’s weight and MUAC increased.

**Table 1.** OptiMA-DRC trial design

	Standard Protocol		OptiMA Protocol		
	MAM	SAM	Acute malnutrition		
Wasting definition	MUAC 115–124 mm or −3 < WHZ < −2	MUAC < 115 mm or WHZ < −3 (SD) or bipedal oedema	MUAC < 125 mm or bipedal oedema		
Treatment product and quantity	RUSF	RUTF	RUTF		
	one 92 g sachet /d (500 kcal/d)	150–200 kcal/kg/d	170−200 (kcal/kg/d)^a^for MUAC < 115 mm or bipedal oedema	125−190 (kcal/kg/d) for MUAC 115−119 mm	50−166 (kcal/kg/d) for MUAC 120−124 mm
Calculation of dosage	fixed amount, regardless of weight or MUAC status	According to the weight	According to MUAC status and weight		

a Based on Cazes *et al*. (2020). kcal: kilocalories corresponding to the dosage of RUTF/RUSF. kg: kilogram of child’s weight. d: day. kcal/kg/d describes the dosage amount (kcal) per each unit of child’s weight (kg) per day (d).

RUTF and RUSF supplementation in both the standard and OptiMA groups was stopped once children reached the recovery state, defined as achieving MUAC ≥ 125 mm in OptiMA or, MUAC ≥ 125 mm or WHZ ≥ −1.5 in the standard group without oedema for two consecutive weeks, in good clinical health, i.e. an axillary temperature below 37.5°C, and enrolled in the program for at least 4 weeks.

The progress of children in the trial was monitored weekly at health centers for the duration of supplementation and then via bi-monthly home visits for 6 months after inclusion. MUAC and weight were recorded at each visit and height once per month. Treatment for illnesses such as respiratory infection, diarrhea, and malaria (and the medications prescribed) and the quantity of RUTF sachets provided was also noted. If children were admitted for inpatient care, their health condition, medications, and the quantity of RUTF consumed were recorded daily. The total amount of RUSF from the beginning to the end of the program for each child was recorded.

At study conclusion, children were classified as having either a favorable or unfavorable outcome at 6 months, defined as: child alive, not acutely malnourished, and no additional episode of acute malnutrition (relapse) throughout the 6 month period following inclusion (Cazes et al., 2020). ‘Not acutely malnourished’ was defined as MUAC ≥ 125 mm, WHZ ≥ −3, and absence of bilateral nutritional oedema.

### Cost and cost-effectiveness analysis

Costing of the malnutrition treatment approaches in each of the OptiMA-DRC trial arms was conducted retrospectively using clinical data collected during the trial and administrative records documenting resource use and expenditure, using a bottom-up ingredients approach. Cost estimates include provider and patient costs.

Total treatment cost for each study arm was calculated by multiplying the quantity of each resource used by its unit cost and summing across resource categories. Four broad categories of resources were identified: service utilization, nutritional commodities, general medications, and supply chain. Service utilization included outpatient clinic visits, outpatient home visits, and inpatient clinic care. Costs of outpatient and inpatient visits were calculated based on labor used and other inputs recorded in ALIMA’s expense book. Medications and tests used included vitamin A, mebendazole for deworming, rapid malaria test, and amoxicillin for bacterial infection among SAM children, artemether-lumefantrine (AL) for malaria-positive children, and nystatin for oral candidiasis among SAM children. Price information for medications was obtained from Médecins Sans Frontières (MSF) for 2021–2022.

Costs for supply chain logistics included transportation costs to distribute RUTF/RUSF and medications (local and international freight, vehicular transport to clinics), labor costs for procurement and warehousing, drivers, and office space. The unit price for the supply chain was the total logistic costs divided by the total number of children. The standard arm program uses two distinct supply chains managed by different organizations, one for RUTF and another for RUSF. Because the OptiMA program would only require one supply chain, we assumed a proportional reduction in cost of 35% due to consolidation of warehouse space, reduced complexity of inventory management, and fewer deliveries. We tested this assumption in sensitivity analysis.

The main outcome metric for our cost-effectiveness analysis is the incremental cost-effectiveness ratio (ICER), expressed as the cost required to obtain an additional successfully treated patient. We conducted t-tests to compare the total cost per child enrolled and effectiveness between the OptiMA group and the standard group and reported *P*-values. We reported the mean, median, and interquartile range (ICR) of costs by looking at the distribution of costs estimates. Where an intervention had equal or better effectiveness and lower cost than a comparator, it was considered dominant and an ICER was not computed. Instead, we reported the magnitude of cost savings per patient treated. When an intervention was dominant, we also calculated the average cost effectiveness ratio (ACER) of each strategy and reported the cost difference per successfully treated patient

During the trial, most of the patients (62%) in the standard arm with a diagnosis of MAM at enrollment did not receive MAM treatment per protocol due to stockouts of RUSF. For this reason, we examined cost-effectiveness overall and separately for patients enrolled with SAM and MAM.

We conducted three sensitivity analyses. First, we conducted probabilistic sensitivity analysis, which simultaneously considered the uncertainties in the quantities of resources used based on observed variation across patients in the trial. We used bootstrapping methods (Obenchain 1999) to construct 10 000 simulated datasets by sampling from the original trial data with replacement. For each simulated dataset, we computed the mean and median cost for OptiMA and the national protocol, as well as the portion of cases successfully treated. From this analysis, we calculated the probability that OptiMA is cost-saving and the probability that OptiMA is cost-effective, as compared to the standard care. Second, we explored how the magnitude of assumed savings on supply chain logistics affected overall cost and cost-effectiveness. Third, we evaluated variations in the unit costs for outpatient visits, home visits, inpatient stays, and supply chain logistics in one-way sensitivity analysis.

The probability an AM treatment intervention is cost-effective depends not only on its incremental costs and treatment effectiveness, but also on how much an additional unit of benefit is worth. To analyze this, we used a cost-effectiveness acceptability curve to show the probability that OptiMA is cost-effective as a function of willingness-to-pay. Since benchmark thresholds for cost-effectiveness in resource-limited settings are usually measured in cost per disability-adjusted life years (DALYs) averted, we converted AM treatment success to DALYs averted. Previous studies of similar AM treatment interventions reported averting 1.2 to 5.4 DALYs per child successfully treated (Goudet et al. 2018), (Shekar et al. 2016), (Bachmann 2009, Wilford et al. 2012, Jenkins 2013), (Puett et al. 2013). In our ICER calculations we therefore explored a range of 0–5 DALYs averted per successfully treated case.

Benchmark thresholds for cost-effectiveness in low-income countries typically range from 0.25 to 1.0 times GDP per capita per DALY averted (Woods et al. 2016, Edoka and Stacey 2020). Since DRC’s GDP per capita is $560 (World Bank, World Development Indicators), we considered the threshold for cost-effectiveness in DRC to be between $140 and $560 per DALY averted.

Data analysis was conducted using Stata SE15.1.

## Results

Table 2 presents the unit price for each of the five broad categories of resources. The unit price was $3.45 for an outpatient clinic visit, $5.13 for a home visit, and $34.72 for an inpatient day (Appendix 1). The unit price of RUTF and RUSF was $0.28 and $0.26 per sachet respectively, excluding transportation cost (Cazes et al. 2022). The unit price for supply chain per child enrolled was $13.83. Appendix 2 presents a breakdown of supply chain costs.

**Table 2.** Unit cost for utilization of services, commodities, and resources (US dollars, 2020)

Category		Unit price in US dollars (2020)		Data source
Service Utilization				
	Outpatient clinic visits	3.45		ALIMA’s accounting records in 2021; authors’ calculation (Appendix 1)
	Outpatient home visits	5.13		ALIMA’s accounting records in 2021; authors’ calculation (Appendix 1)
	Inpatient visit cost per day	34.72		ALIMA’s accounting records in 2021; authors’ calculation (Appendix 1)
Nutritional commodities				
	RUTF (one sachet)	0.28		Cazes et al. (2022) (Appendix 1)
	RUSF (PlumpySup, one sachet)	0.26		Cazes et al. (2022) (Appendix 1)
Medications				
Vitamin A		0.05		MSF - Médecins Sans Frontières (MSF) supply (2022)
Deworming	Anthelmintic—mebendazole 100 mg	0.06		MSF supply (2022)
Malaria diagnosis	A rapid malaria test	0.7		MSF supply (2022)
Bacterial infection	Amoxicillin (50–100 mg/kg/day for 7 days) among SAM (no. packet, 1 packet = 250 mg)	0.77		MSF supply (2022)
Malaria treatment	Artemisinin-based combination therapy—AL [artemether−lumefantrine (Coartem, Riamet)] 20/120 mg 5−14 kg	0.28		MSF supply (2022)
Oral candidiasis	Nystatin, 100 000 IU/ml, oral suspension among 20% of SAM treated	0.56		MSF supply (2022)
Supply chain		Standard	OptiMA	
	Supply chain per child	13.83	9.1	ALIMA’s accounting records in 2021; authors’ calculation (Appendix 2)

Table 3 provides the summary statistics of children in the sample. At inclusion, the mean age was 20.1 months (SD: 12.7). The numbers of girls and boys were nearly equal. The mean MUAC was 118 mm (SD:6.3) and 8.7% (78/896) had oedema. The mean weight and height were 7.6 kg (SD: 1.8) and 73.4 cm (SD: 9.4), respectively. About 19% of children (170 /896) had WHZ < −3.

**Table 3.** Baseline characteristics^a^

	Total		SAM at enrollment		MAM at enrollment	
	Standard	OptiMA	Standard	OptiMA	Standard	OptiMA
	*n* = 446	*n* = 450	*n* = 200	*n* = 198	*n* = 246	*n* = 252
Socio-demographic characteristics						
Male	225 (50%)	221 (49%)	101 (50%)	99 (50%)	123 (50%)	122 (48%)
Female	221 (50%)	229 (51%)	99 (50%)	99 (50%)	122 (50%)	130 (52%)
Age (months)	20.6 (12.7)	19.6 (12.7)	21.7 (14.3)	20.2 (13.2)	19.7 (11.2)	19.1 (12.3)
Anthropometric characteristics						
MUAC (mm)	118 (6.2)	118 (6.3)	115 (7.8)	115 (8.2)	121 (2.5)	120 (2.7)
Nutritional oedema	43 (10%)	35 (8%)	43 (22%)	35 (18%)	0 (0%)	0 (0%)
Weight (kg)	7.7 (1.8)	7 · 6 (1.8)	7.4 (2.1)	7 · 3 (2.0)	7.9 (1.5)	7 · 8 (1.7)
Height (cm)	73.6 (9.2)	73.2 (9.6)	73.8(10.8)	72.9 (10.3)	73.6 (7.8)	73.5 (9.1)
WHZ < −3	83 (18.6%)	87 (19.3%)	83 (41.5%)	87 (43.9%)	0 (0%)	0 (0%)

a Data are mean or *n* (%); standard deviations in parentheses.

Table 4 presents the summary statistics on the quantity of resources used. Children enrolled in the OptiMA arm had a mean of 7.9 clinic visits (SD: 4.4), vs. 5.3 (SD: 5.1) in the standard arm. Among children initially enrolled with SAM, the number of clinic visits was similar (8.73 (SD: 4.80) in standard, 8.54 (SD: 4.53) in OptiMA.

**Table 4.** Quantity of resources used per patient (mean)

	Total		SAM at enrollment		MAM at enrollment	
	Standard (*n* = 446)	OptiMA (*n* = 450)	Standard (*n* = 200)	OptiMA (*n* = 198)	Standard (*n* = 246)	OptiMA (*n* = 252)
Outpatient visits per patient (number)						
Clinic visits	5.34	7.89	8.73	8.54	2.59	7.37
Home visits	8.84	7.49	7.19	6.86	10.18	7.98
Inpatient hospitalization						
Probability	0.072	0.096	0.125	0.116	0.028	0.079
Days, per patient hospitalized	7.6	7.1	7.5	6.0	8.0	8.3
Days, per patient	0.55	0.68	0.94	0.70	0.23	0.65
RUTF per patient (sachets)	96.55	82.30	181.29	99.46	27.66	68.82
RUSF (sachets)						
Per patient	5.77	not applicable (na)	0.00	na	10.46	na
Per patient receiving >0 sachet	27.38	na	0.00	na	27.38	na
Treated comorbidity per patient						
Vitamin A	1	1	1	1	1	1
Deworming	1	1	1	1	1	1
Malaria diagnosis	1	1	1	1	1	1
Bacterial infection	0.45	0.44	1	1	0	0
Malaria treatment	0.76	0.77	0.75	0.74	0.76	0.79
Oral candidiasis	0.09	0.09	0.20	0.20	0.00	0.00

Home visits were conducted 8.8 times (SD: 3.0) for children for the standard arm and 7.5 times (SD: 2.6) for the OptiMA arm. A total of 7% (32/446) and 10% (43/450) of patients were hospitalized in the standard and OptiMA arms, respectively. When hospitalization did occur, the duration was 7.6 days (SD: 4.3) for standard and 7.1 days (SD: 4.4) for OptiMA.

For patients initially enrolled with SAM, the mean amount of RUTF distributed per child was 181 sachets (SD: 101) in the standard group and 99 sachets (SD: 60) for OptiMA. In OptiMA, patients enrolled with MAM received a mean of 69 RUTF sachets (SD: 46). Patients in the standard arm were supposed to receive RUSF, but lack of continuous supply resulted in only 36% (88/246) receiving any RUSF. Among this subset of patients, the mean was 27.3 sachets (SD: 10.5).

In the standard arm, the malnutrition status of 40/246 (16%) children with MAM at inclusion worsened to the point of qualifying for SAM treatment. They were treated with RUTF at an average of 170.1 sachets per child (mean). In contrast, only 12 of 252 (5%) of patients enrolled in the OptiMA arm with MAM worsened to SAM.

All children received vitamin A, deworming medication, and malaria diagnostic tests, and those categorized as SAM upon inclusion received amoxicillin for bacterial infection and nystatin for oral candidiasis if necessary. A large share of children (77%; 683/896) received malaria treatment in both arms.

Overall, there was no significant difference in the total cost per child enrolled at $126.5 and $122.6 (*P* = 0.55) in the standard and OptiMA arms, respectively (Table 5). However, all children in OptiMA and those enrolled with SAM in the standard group received RUTF, whereas only 38% of those enrolled with MAM in the standard group received the indicated RUSF, due to stock outs. The remaining 62% did not receive RUSF or other treatment. When restricting the analysis to patients enrolled with SAM, fewer resources were used in every category for the OptiMA group compared to the standard arm, with total cost per child 23% lower with OptiMA ($166.1 standard vs $127.9 OptiMA). Among children enrolled with MAM, the total cost was 25% higher with OptiMA ($94.7 standard vs $118.5 OptiMA).

**Table 5.** Cost per child enrolled (US dollars, 2020)

	Standard		OptiMA		SAM at enrollment				MAM at enrollment			
	(*n* = 446)		(*n* = 450)		Standard		OptiMA		Standard		OptiMA	
					(*n* = 200)		(*n* = 198)		(*n* = 246)		(*n* = 252)	
	mean	median	mean	median	mean	median	mean	median	mean	median	mean	median
Outpatient visits												
Clinic visits	18.42	17.25	27.20	20.7	30.10	24.15	29.47	24.15	8.93	3.45	25.44	20.7
Home visits	45.32	46.15	38.39	41.02	36.84	41.02	35.17	35.89	52.22	56.41	40.92	41.02
Hospitalization												
Per patient enrolled	18.99	0	23.47	0	32.81	0	24.37	0	7.87	0	22.73	0
Per patient hospitalized	264.7		245.43		261.06		209.68		277.72		286.4	
RUTF	27.04	28.42	23.04	17.92	50.76	41.16	27.85	22.26	7.75	0	19.27	14.84
RUSF	1.49	0	not applicable (na)	na	0	0	na	na	2.71	0	na	na
Comorbidity treatment	1.42	1.09	1.42	1.09	1.91	1.98	1.91	1.98	1.03	1.09	1.03	1.09
Local supply chain	13.83	13.83	9.1	9.1	13.83	13.83	9.1	9.1	13.83	13.83	9.1	9.1
Total	126.52	103.37	122.62	95.55	166.08	123.54	127.86	98.42	94.37	79.91	118.49	91.60
	95% CI	IQR	95% CI	IQR	95% CI	IQR	95% CI	IQR	95% CI	IQR	95% CI	IQR
	[117.48–135.57]	79.63; 127.92	[113.55–131.68]	87.03; 110.36	[150.14–182.03]	114.77; 163.29	[115.76–139.96]	92.49; 122.45	[86.19–102.54]	73.54; 87.67	[105.33–131.65]	85.63; 103.73

In the trial, OptiMA was significantly more effective than the standard national protocol at producing recovered patients without relapse at 6 months. Patients in the OptiMA arm had a 9% higher treatment success rate (72 vs 63%). Since OptiMA was no more expensive than the standard care, but was more effective, OptiMA was the dominant strategy (Table 6). A comparison of the ACER for each strategy showed that OptiMA cost $31 less per successful outcome.

**Table 6.** Cost-effectiveness results^a^

Panel A: total sample					
	Standard (*n* = 446)	OptiMA (*n* = 450)	Difference	Significance, *P*-value (difference)	ICER
(Cost per Success)					
Total cost per child enrolled (USD, 2020)	126.52	122.63	−3.89	0.549	
Effectiveness (success rate)	0.63	0.72	0.09	0.0039	OptiMA is dominant
95% CI	[0.59–0.68]	[0.68–0.76]			
ACER^b^ ($ per success)	200.82	170.31	−30.51		
Panel B: initial SAM					
	Standard (n = 200)	OptiMA (n = 198)	Difference	*P*-value	ICER
(Cost per Success)					
Total cost per child enrolled (USD, 2020)	166.06	127.86	−38.21	<0.0001	
Effectiveness (success rate)	0.62	0.70	0.07	0.13	OptiMA is dominant
95% CI	[0.56–0.69]	[0.63–0.76]			
ACER ($ per success)	267.84	182.65	−85.19		
Panel C: initial MAM					
	Standard (*n* = 246)	OptiMA (*n* = 252)	Difference	*P*-value	ICER
(Cost per progression averted)					
Total cost per child enrolled (USD, 2020)	94.67	118.49	24.82	0.0022	
Effectiveness (progression to SAM)	0.16	0.05	−0.11	<0.0001	$234
95% CI	[0.58–0.70]	[0.69–0.80]			

a Cost is for one additional successfully treated child. Success is defined as being alive, not acutely malnourished, without relapse during the 6 month period after the initiation of the program.

b ACER, reported in place of ICER, to calculate the cost saving per successfully treated patient when an intervention was dominant.

OptiMA did not categorize patients into SAM and MAM, but because the standard arm did, it can be useful to analyze outcomes for these groups separately to better understand the drivers of cost-effectiveness. Among children enrolled with SAM, OptiMA was the dominant strategy and was no less effective (0.70 vs 0.62; *P* < 0.12) and $38.21 less expensive per enrolled patient than the standard (*P* < 0.0001). A comparison of the ACER for each strategy showed that OptiMA cost $85 less per successful outcome.

Among children enrolled with MAM, the incidence of children deteriorating to SAM (average 74 days) in the standard group was more than three times higher than with OptiMA (16 vs 5%; *P* < 0.0001). While OptiMA was more effective at preventing progression to SAM, it was more expensive, even after accounting for the additional cost of treating SAM among those patients with progression, due in large part to the fact that 64% (159/246) of standard arm patients enrolled with MAM received no RUSF treatment. Among patients enrolled with MAM, OptiMA cost an additional $234 for each case of progression to SAM prevented as compared to the standard arm.

### Sensitivity analysis

Probabilistic sensitivity analysis was conducted to account for uncertainty in the resource use and treatment success probabilities. Results (Fig. 1) show that there is a 99% chance that OptiMA is more effective than the status quo implementation of the national protocol and a 71% chance that it is also cost-saving. Additionally, there is a 92% chance that the median cost of OptiMA is lower than the median cost of the national protocol.

**Figure 2.**
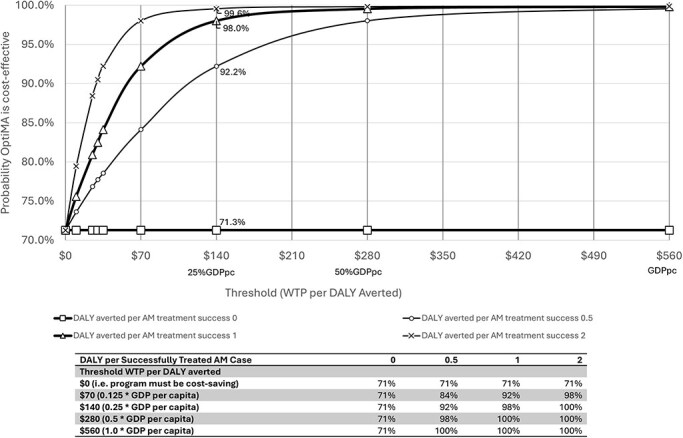
Cost-effectiveness acceptability curves for different assumptions about the DALYs averted per successfully treated acute malnutrition case.

If OptiMA were not cost-saving, the probability that it is cost-effective would depend on the threshold for good value—i.e. how many DALYs are averted by successfully treating a case of AM, and what that health benefit is worth. The cost-effectiveness acceptability curve shown in Fig. 2 indicates that even with as little as 0.5 DALY averted per successfully treated AM case the OptiMA strategy is very likely to be cost-effective compared to the status quo national protocol, with a 98% chance of having an ICER less than the cost-effectiveness threshold of $140 (25% of GDP per capita) per DALY averted. In one-way sensitivity analysis, we varied the mean costs of clinic visit, home visit, and hospitalization between 50 and 200% of the base case (Appendix 3). We also tested the relative efficiency of supply chain costs, using zero efficiency gain at one extreme (supply chain costs the same in OptiMA and standard) (Appendix 4) and 50% efficiency gain at the other extreme. Results of OptiMA’s dominance or cost-effectiveness using different scenarios as part of sensitivity analyses did not change from the base case. Out of eight scenarios, five still concluded that OptiMA was the dominant strategy. If the cost of clinic visits was assumed to be twice those in our base case, OptiMA became more expensive but still cost-effective, with an ICER of $57 per successfully treated case, assuming that successfully treating a case results in at least 0.5 DALY averted. If supply chain cost was identical for OptiMA and standard, then the total cost per child enrolled was only slightly higher under OptiMA ($123.06 vs $122.09), and the ICER for OptiMA vs standard was $10.7 per successfully treated case.

## Discussion

Given the heavy burden of acute malnutrition in children in sub-Saharan Africa and South Asia, financing shortfalls, and constrained supply of therapeutic foods, there is a pressing need to find more efficient treatment approaches. The results of this analysis indicate that OptiMA is likely one such approach. In the DRC trial, OptiMA was the dominant strategy compared to the status quo, because it was more effective and no more costly. When focusing on patients enrolled with SAM, we found that the OptiMA approach was no less effective and reduced cost by 23%, saving $38 per person treated. Additionally, the probability of deteriorating from MAM to SAM in OptiMA was lower compared to the standard national protocol for DRC.

Our analysis shows that the mean cost per child enrolled in the OptiMA and standard groups was similar at $123 and $127, respectively. OptiMA’s unified approach to treating all severity levels of acute malnutrition resulted in a similar overall cost per child enrolled as the standard approach, but the allocation of resources across patients was different in the two arms. The OptiMA group had higher costs for clinic visits while the standard group had higher costs for home visits because the OptiMA was functioning at all times and patients were more likely to comply with the intervention and come back for a clinic visit for follow-up, while the standard approach had supply-side challenges and children were more likely to be followed-up at their homes. The OptiMA group had higher hospitalization costs per child enrolled but lower costs on RUTF.

The OptiMA program costs less than the standard approach per child enrolled with SAM, but more per child enrolled with MAM. The result among SAM patients is promising but will benefit from further studies to replicate this finding. When focusing on children enrolled with SAM, the mean cost for the OptiMA and standard groups was $128 and $166, respectively, largely due to the lower quantity of RUTF per patient under OptiMA. These findings are similar to those in other studies using shorter follow-up times, with a mean cost per child ranging from $166–314 in 2020 USD for the treatment of AM [reported in Njuguna et al. 2020). (Mean cost per child of $166 in 2020 USD in Uganda (Jenkins 2013), $216 to 248 in 2020 USD in India (Menon et al. 2016, Garg et al. 2018), and $292 in 2020 USD in Indonesia (Purwestri et al. 2012); community-based treatment cost of $168 in 2020 USD per child in Ethiopia (Tekeste et al. 2012), $196 in 2020 USD in Bangladesh (Puett et al. 2013), 149 euros (or $220 in 2020 USD) in Niger (Isanaka et al. 2017), $226 in 2020 USD in Zambia (Bachmann 2009), and $314 in 2020 USD in Pakistan (Rogers et al. 2019). These studies mainly focused on SAM children, except for Jenkins (2013) which included both SAM and MAM and Purwestri et al. (2012) which focused on MAM.]

In our study, among patients enrolled with MAM, OptiMA provided treatment to a much larger portion of patients, was more effective at producing recovered patients without relapse at 6 months, and greatly reduced risk of progression to SAM. Although the standard approach saved resources by leaving untreated more than half of the children enrolled with MAM, this had negative consequences as shown through the higher rate of deterioration to SAM (16% with the standard approach vs 5% with OptiMA).

Improved outcomes for MAM patients are an important benefit of the OptiMA approach. Previous studies have estimated that children with MAM have 2.5 times higher mortality than well-nourished children after discharge from hospital (Diallo et al. 2022), and are more susceptible to diseases that cause substantially higher risk of mortality and morbidity. Treatment of MAM reduces the chance of relapses, deterioration to SAM, and death (Chang et al. 2013).

Limitations of our study include a small sample size and the lack of long-term follow-up to observe the cumulative impact and broader set of long-term consequences of programs for treating AM episodes that may recur through childhood, although the follow-up period in this study is longer than in many other studies. Another limitation is the poor adherence to the national protocol, resulting in lack of treatment, among more than half of MAM children under the standard group. We do not know what the cost or effectiveness of the national protocol would have been if the MAM programme had been functioning with a stable supply of RUSF. However, our results capture the real-world implementation challenges facing such programs. Uncertainty regarding the impact of adopting OptiMA on the efficiency of the supply chain is a further limitation. In the context of a single randomized control trial, we could not measure the amount of cost-savings that might accrue from consolidating supply chains in a large-scale program with RUTF used as the only therapeutic food product for all patients. However, even if our assumption of a 35% reduction in supply chain cost turns out to be too large, our conclusions from this study are not sensitive to this uncertainty. Assuming no gains in supply chain efficiency from eliminating the RUSF supply channel, OptiMA would still have a highly favorable ICER of $9 per additional case successfully treated.

OptiMA had significantly better outcomes and was no more expensive than standard care. Its adoption could enable more acutely malnourished children to be successfully treated in contexts where therapeutic food products are scarce.

## Supplementary Material

czae106_Supp

## Reflexivity statement

We have a wide range of authors in terms of demographic characteristics with various expertise. We have 6 females and 13 male coauthors. Some of us are medical doctors, others are economists, decision scientists, nutritionists, statisticians, and epidemiologists. All authors have extensive experience of conducting research in developing countries, especially in West Africa.

## Data availability

The data underlying this article cannot be shared publicly due to the confidentiality of individuals that participated in the study. The data will be shared on reasonable request to the corresponding author.

**Appendix 1.** Breakdown of unit cost (US dollars, 2020)

	Cost per child enrolled, US dollars 2020		Notes
Panel A1: outpatient clinic visits			
Labor costs (outpatient) per visit	1.57		$352 470/(115 309 consultations for SAM children + 109 091 general consultations for children < 5 years old = 224 400 visits during the 30 month study period)
Non-labor costs (outpatient)	1.15		$8590/month x 30 months = $257 700/224 400 visits (from above). Non-labor costs included office-running costs, utilities, vehicle operation, and supplies.
Opportunity costs for outpatient visits	0.06		The average time for an outpatient visit under the OptiMA arm is 20 min.
Transportation costs (opportunity costs)	0.67		Transportation costs were opportunity costs of travel time for the caregiver. Most children and caregivers visit the clinic on foot, it takes 3.5 h on average for transportation. Thus, we used the GDP per capita of $560 divided by 365 days/year divided by 8 h/day = $0.192 per hour x 3.5 h.
Total cost of outpatient clinic visit per child enrolled	3.45		
Panel A2: outpatient home visits			
Labor costs (home visit)	3.12		$36 414 for 4 nurses, 75% used for 8740 home visits for 1071 children
Transportation costs (home visits)	2.00		$17 505 for motorcycle-related costs (fuel, maintenance, purchase, drivers) for 8740 home visits to 1071 children
Total cost of outpatient home visit per child enrolled	5.13		
Panel B: RUTF/RUSF			
RUTF (1 sachet)	0.28		$42 for 150 sachets of RUTF
RUSF (PlumpySup, 1 sachet)	0.26		$38.83 for 150 sachets of RUSF
Panel C: inpatient visits			
Labor costs (inpatient) per day	23.68		$437 325 for 30 months/(2530 children for 7.3 days), 7.3 days is from the data. Labor costs include costs for ALIMA and Ministry of Health.
Medications/supplies	9.51		Cost in this category was calculated based on clinician expert opinion regarding the quantity of medicines and supplies (such as antibiotics, needle, syringe, nasogastric tube, oral solution for treatment of dehydration etc.) consumed by a typical inpatient case of acute malnutrition. Unit cost of each item was obtained from ALIMA’s accounting records.
Opportunity costs per day	1.53		GDP per capita of $560 divided by 365 days
Total cost of stay per day per child hospitalized	34.72		
Panel D: supply chain	Control	OptiMA	
Total cost of supply chain per child enrolled	13.83	9.1	See Appendix 4 for the breakdown. We assume that the supply chain for RUTF costs the same as the supply chain for RUSF. Because supply chain under control deals with both RUTF and RUSF, it is more expensive than the supply chain cost under OptiMA.

**Appendix 2.** Breakdown of supply chain costs (US dollars, 2020)

		Cost per child		
Category	Total	OptiMA	Control	Assumptions
Local and international freight—medications	3072	0.18	0.18	Same cost between OptiMA and control; $153 600 for freight of all medications, 2% of which were used for malnourished children.
Vehicles (for distributing medication to clinics)	2655	0.15	0.15	Same cost between OptiMA and control; $132 750 for vehicles for all medications, 2% of which were used for malnourished children
Labor (procurement, warehouse workers, drivers, etc.)	31 918	1.87	3.75	Cost per child under control is double the cost under OptiMA for two parallel supply chains
Local and international freight RUTF	90 878	5.34	6.63	Shipping cost is proportional to the number of sachets
Vehicles (for distributing RUTF to clinics)	7014	0.41	0.82	Cost under control is double the cost under OptiMA because of the vehicles for two parallel supply chain
Warehouse rent	19 790	1.15	2.29	Cost per child under control is double the cost under OptiMA for two parallel supply chains. Warehouse is for all supplies for all programs, including OptiMA and other programs
Total	155 328	9.10	13.83	

**Appendix 3.** Sensitivity analysis—cost variation

	Control (*n* = 446)	OptiMA (*n* = 450)	Difference	Significance, *P*-value (difference)	ICER
Total cost per child enrolled (USD, 2020)—default	126.52	122.62	−3.89	0.55	-
50% costs					
Total cost per child enrolled (USD, 2020)—clinic visit cost 50%	117.31	109.01	−8.30	0.18	-
Total cost per child enrolled (USD, 2020)—home visit cost 50%	103.86	103.42	−0.44	0.95	-
Total cost per child enrolled (USD, 2020)—hospitalization cost 50%	117.03	110.89	−6.14	0.13	-
Total cost per child enrolled (USD, 2020)—supply chain cost same (Control = Treatment [OPtiMA])	121.79	122.62	0.82	0.90	9.12
200% costs					
Total cost per child enrolled (USD, 2020)—clinic visit cost 200%	144.95	149.83	4.87	0.50	54.20
Total cost per child enrolled (USD, 2020)—home visit cost 200%	171.85	161.01	−10.84	0.08	-
Total cost per child enrolled (USD, 2020)—hospitalization cost 200%	145.52	146.07	0.55	0.96	6.12
Total cost per child enrolled (USD, 2020)—supply chain cost same (Control = 2 x Treatment [OptiMA])	130.89	122.62	−8.28	0.20	-
Effectiveness (success rate)	0.63	0.72	0.09	0.00	

**Appendix 4.** Sensitivity analysis for no supply chain cost savings scenario: cost estimate and ICER

	Total		SAM at enrollment		MAM at enrollment	
	Control (*n* = 446)	OptiMA (*n* = 450)	Control (*n* = 199)	OptiMA (*n* = 198)	Control (*n* = 247)	OptiMA (n = 252)
Outpatient visits						
Clinic visits	18.42	27.20	29.97	29.46	9.12	25.43
Home visits	45.32	38.39	36.88	35.17	52.13	40.92
Hospitalization						
Per patient enrolled	18.99	23.47	32.81	24.37	7.87	22.73
RUTF	27.04	23.04	50.67	27.85	7.99	19.27
RUSF	1.49	0.00	0.00	0.00	2.70	0.00
Comorbidity treatment						
Vitamin A	0.05	0.05	0.05	0.05	0.05	0.05
Deworming	0.06	0.06	0.06	0.06	0.06	0.06
Malaria diagnosis	0.70	0.70	0.70	0.70	0.70	0.70
Bacterial infection	0.35	0.34	0.77	0.77	0.00	0.00
Malaria treatment	0.21	0.21	0.21	0.21	0.21	0.22
Oral candidiasis	0.05	0.05	0.11	0.11	0.00	0.00
Local supply chain	9.10	9.10	9.10	9.10	9.10	9.10
Total cost per child enrolled (USD, 2020)	121.79	122.63	161.33	127.86	89.94	118.49
Effectiveness (success rate)	0.63	0.72	0.62	0.70	0.64	0.74
ICER		9.33		OptiMA is dominant		279.89

Note: This sensitivity analysis assumes the supply chain cost is the same under control and OptiMA programs.
