# Supplementary material for: Cost-effectiveness of a simplified acute malnutrition program: a secondary analysis of the OptiMA randomized clinical trial in the Democratic Republic of the Congo
Source: Health Policy Plan. 2024 Nov 8;40(3):273–86. doi: 10.1093/heapol/czae106 (PMC11886841; doi:10.1093/heapol/czae106)
Supplement: czae106_Supp [file czae106_supp.zip › Supplementary/Supplementary.docx]

| Appendix 1: Breakdown of Unit Cost (US dollars, 2020) | | | |
| --- | --- | --- | --- |
|  | Cost per child enrolled, US dollars 2020 | | Notes |
|  |  |  |  |
| Panel A1: Outpatient clinic visits |  |  |  |
| labor costs (outpatient) per visit | 1.57 |  | “$352,470/ (115,309 consultations for SAM children + 109,091 general consultations for children under 5 = 224,400 visits during the 30-month study period) |
| non labor costs (outpatient) | 1.15 |  | $8590/ month * 30 months = $257,700 / 224,400 visits (from above). Non-labor costs included office-running costs, utilities, vehicle operation and supplies. |
| opportunity costs for outpatient visits | 0.06 |  | The average time for outpatient visit under OptiMA arm is 20 minutes. |
| transportation costs (opportunity costs) | 0.67 |  | Transportation costs were opportunity costs of travel time for the caregiver. Most children and caregivers visit the clinic on foot, it takes 3.5 hours on average for transportation. Thus, we used the GDP per capita of $560 divided by 365 days/yr divided by 8 hours/day = $0.192 per hour * 3.5 hours. |
| Total cost of outpatient clinic visit per child enrolled | 3.45 |  |  |
| Panel A2: Outpatient home visits |  |  |  |
| labor costs (home visit) | 3.12 |  | $36,414 for 4 nurses, 75% used for 8740 home visits for 1071 children |
| transportation costs (home visits) | 2.00 |  | $17,505 for motorcycle-related costs (fuel, maintenance, purchase, drivers) for 8740 home visits to 1071 children |
| Total cost of outpatient home visit per child enrolled | 5.13 |  |  |
| Panel B: RUTF / RUSF |  |  |  |
| RUTF (1 sachet) | 0.28 |  | $42 for 150 sachets of RUTF |
| RUSF (PlumpySup, 1 sachet) | 0.26 |  | $38.83 for 150 sachets of RUSF |
| Panel C: Inpatient visits |  |  |  |
| labor costs (inpatient) per day | 23.68 |  | $437,325 for 30 months / (2530 children for 7.3 days), 7.3 days is from the data. Labor costs include costs for ALIMA and MoH. |
| medications / supplies | 9.51 |  | Cost in this category was calculated based on clinician expert opinion regarding the quantity of medicines and supplies (such as antibiotics, needle, syringe, nasogastric tube, oral solution for treatment of dehydration etc.) consumed by typical inpatient case of acute malnutrition. Unit cost of each item was obtained from ALIMA’s accounting records. |
| opportunity costs per day | 1.53 |  | GDP per capita of $560 divided by 365 days |
| Total cost of stay per day per child hospitalized | 34.72 |  |  |
| Panel D: Supply chain | Control | OptiMA |  |
| Total cost of supply chain per child enrolled | 13.83 | 9.1 | See Appendix 3 for the breakdown. We assume that the supply chain for RUTF costs the same as the supply chain for RUSF. Because supply chain under control deals with both RUTF and RUSF, it is more expensive than the supply chain cost under OptiMA. |
| Note: Aggregated costs and quantities indicated in Notes are based on ALIMA’s accounting records in 2021. Costs reported in Euro were converted to dollars at the 2020 exchange rate1euro=1.142USD. | | | |

| Appendix 2: Breakdown of Supply Chain Costs (US dollars, 2020) | | | | |
| --- | --- | --- | --- | --- |
| Category | Total | Cost per child | | Assumptions |
|  |  | OptiMA | Control |  |
| Local& International freight – medications | 3072 | 0.18 | 0.18 | Same cost between OptiMA and control; $153,600 for freight of all medications, 2% of which were used for malnourished children. |
| Vehicles (for distributing medication to clinics) | 2655 | 0.15 | 0.15 | Same cost between OptiMA and control; $132,750 for vehicles for all medications, 2% of which were used for malnourished children |
| Labor (procurement, warehouse workers, drivers, etc) | 31918 | 1.87 | 3.75 | cost per child under control is double the cost under OptiMA for 2 parallel supply chain |
| Local & International freight RUTF | 90878 | 5.34 | 6.63 | shipping cost is proportional to the number sachet |
| Vehicles (for distributing RUTF to clinics) | 7014 | 0.41 | 0.82 | cost under control is double the cost under OptiMA because of the vehicles for 2 parallel supply chain |
| Warehouse rent | 19790 | 1.15 | 2.29 | cost per child under control is double the cost under OptiMA for 2 parallel supply chain. Warehouse is for all supplies for all programs, including OptiMA and other programs |
| Total | 155328 | 9.10 | 13.83 |  |

| Appendix 3: Sensitivity Analysis for No Supply Chain Cost Savings Scenario: Cost estimate and ICER | | | | | | | | |
| --- | --- | --- | --- | --- | --- | --- | --- | --- |
|  | Total | |  | SAM at enrollment | |  | MAM at enrollment | |
|  | Control (n=446) | OptiMA (n=450) |  | Control (n=199) | OptiMA (n=198) |  | Control (n=247) | OptiMA (n=252) |
| Outpatient visits |  |  |  |  |  |  |  |  |
| clinic visits | 18.42 | 27.20 |  | 29.97 | 29.46 |  | 9.12 | 25.43 |
| home visits | 45.32 | 38.39 |  | 36.88 | 35.17 |  | 52.13 | 40.92 |
| Hospitalization |  |  |  |  |  |  |  |  |
| Per patient enrolled | 18.99 | 23.47 |  | 32.81 | 24.37 |  | 7.87 | 22.73 |
| RUTF | 27.04 | 23.04 |  | 50.67 | 27.85 |  | 7.99 | 19.27 |
| RUSF | 1.49 | 0.00 |  | 0.00 | 0.00 |  | 2.70 | 0.00 |
| Comorbidity treatment |  |  |  |  |  |  |  |  |
| *Vitamin A* | 0.05 | 0.05 |  | 0.05 | 0.05 |  | 0.05 | 0.05 |
| *Deworming* | 0.06 | 0.06 |  | 0.06 | 0.06 |  | 0.06 | 0.06 |
| *Malaria diagnosis* | 0.70 | 0.70 |  | 0.70 | 0.70 |  | 0.70 | 0.70 |
| *Bacterial infection* | 0.35 | 0.34 |  | 0.77 | 0.77 |  | 0.00 | 0.00 |
| *Malaria treatment* | 0.21 | 0.21 |  | 0.21 | 0.21 |  | 0.21 | 0.22 |
| *Oral candidiasis* | 0.05 | 0.05 |  | 0.11 | 0.11 |  | 0.00 | 0.00 |
| Local supply chain | 9.10 | 9.10 |  | 9.10 | 9.10 |  | 9.10 | 9.10 |
| Total cost per child enrolled (USD, 2020) | 121.79 | 122.63 |  | 161.33 | 127.86 |  | 89.94 | 118.49 |
| Effectiveness (success rate) | 0.63 | 0.72 |  | 0.62 | 0.70 |  | 0.64 | 0.74 |
| ICER |  | 9.33 |  |  | OptiMA is dominant |  |  | 279.89 |
| Notes: This sensitivity analysis assumes the supply chain cost is the same under control and OptiMA program. | | | | | | | | |

Appendix 4: Sensitivity Analysis – Cost variation

|  | Control (n=446) | OptiMA (n=450) | Difference | significance, p-value (difference) | ICER |
| --- | --- | --- | --- | --- | --- |
| Total cost per child enrolled (USD, 2020) - default | 126.52 | 122.62 | -3.89 | 0.55 | - |
| 50% costs |  |  |  |  |  |
| Total cost per child enrolled (USD, 2020) - clinic visit cost 50% | 117.31 | 109.01 | -8.30 | 0.18 | - |
| Total cost per child enrolled (USD, 2020) - home visit cost 50% | 103.86 | 103.42 | -0.44 | 0.95 | - |
| Total cost per child enrolled (USD, 2020) - hospitalization cost 50% | 117.03 | 110.89 | -6.14 | 0.13 | - |
| Total cost per child enrolled (USD, 2020) - supply chain cost same (C = T) | 121.79 | 122.62 | 0.82 | 0.90 | 9.12 |
| 200% costs |  |  |  |  |  |
| Total cost per child enrolled (USD, 2020) - clinic visit cost 200% | 144.95 | 149.83 | 4.87 | 0.50 | 54.20 |
| Total cost per child enrolled (USD, 2020) - home visit cost 200% | 171.85 | 161.01 | -10.84 | 0.08 | - |
| Total cost per child enrolled (USD, 2020) - hospitalization cost 200% | 145.52 | 146.07 | 0.55 | 0.96 | 6.12 |
| Total cost per child enrolled (USD, 2020) - supply chain cost same (C = 2*T) | 130.89 | 122.62 | -8.28 | 0.20 | - |
|  |  |  |  |  |  |
| Effectiveness (success rate) | 0.63 | 0.72 | 0.09 | 0.00 |  |
